# Correlation of Cerebral and Subcutaneous Glycerol in Severe Traumatic Brain Injury and Association with Tissue Damage

**DOI:** 10.1007/s12028-021-01412-z

**Published:** 2021-12-16

**Authors:** Linda Hägglund, Magnus Olivecrona, Lars-Owe D. Koskinen

**Affiliations:** 1 Department of Clinical Science, Neurosciences, Umeå University, Umeå, Sweden; 2 Department of Anesthesia and Intensive Care, Section of Neurosurgery, Örebro University Hospital and Department for Medical Sciences, Faculty of Health and Medicine, Örebro University, Örebro, Sweden

**Keywords:** Traumatic brain injury, Glycerol, S-100B, Neuron-specific enolase, Microdialysis, Brain computed tomography, Outcome

## Abstract

**Background:**

This study is a substudy of a prospective consecutive double-blinded randomized study on the effect of prostacyclin in severe traumatic brain injury (sTBI). The aims of the present study were to investigate whether there was a correlation between brain and subcutaneous glycerol levels and whether the ratio of interstitial glycerol in the brain and subcutaneous tissue (glycerol_brain/sc_) was associated with tissue damage in the brain, measured by using the Rotterdam score, S-100B, neuron-specific enolase (NSE), the Injury Severity Score (ISS), the Acute Physiology and Chronic Health Evaluation Score (APACHE II), and trauma type. A potential association with clinical outcome was explored.

**Methods:**

Patients with sTBI aged 15–70 years presenting with a Glasgow Coma Scale Score ≤ 8 were included. Brain and subcutaneous adipose tissue glycerol levels were measured through microdialysis in 48 patients, of whom 42 had complete data for analysis. Brain tissue damage was also evaluated by using the Rotterdam classification of brain computed tomography scans and the biochemical biomarkers S-100B and NSE.

**Results:**

In 60% of the patients, a positive relationship in glycerol_brain/sc_ was observed. Patients with a positive correlation of glycerol_brain/sc_ had slightly higher brain glycerol levels compared with the group with a negative correlation. There was no significant association between the computed tomography Rotterdam score and glycerol_brain/sc_. S-100B and NSE were associated with the profile of glycerol_brain/sc_. Our results cannot be explained by the general severity of the trauma as measured by using the Injury Severity Score or Acute Physiology and Chronic Health Evaluation Score.

**Conclusions:**

We have shown that peripheral glycerol may flux into the brain. This effect is associated with worse brain tissue damage. This flux complicates the interpretation of brain interstitial glycerol levels. We remind the clinicians that a damaged blood–brain barrier, as seen in sTBI, may alter the concentrations of various substances, including glycerol in the brain. Awareness of this is important in the interpretation of the data bedside as well in research.

## Introduction

Traumatic brain injury (TBI) can be described as mild, moderate, or severe (sTBI) and is often classified on the basis of level of consciousness and/or the length of posttraumatic amnesia. sTBI is the main cause of morbidity and mortality among young adults younger than 40 years of age, and the mortality rate is estimated to be 10 per 100,000 inhabitants per year in Sweden [1]. The overall levels of in-patient care of TBI have seemed to decrease, but there is an increase in older adults [2]. A similar trend is reported in other European countries [3]. In a recent review, the global burden, consequences, and health economy costs were found to be very large [4].

Glycerol is a molecular component of triglycerides stored in the human fatty tissue. Triglycerides are decomposed into free glycerol and fatty acids through sympathetic activity. Glycerol does not normally pass the blood–brain barrier (BBB). In the central nervous system, glycerol is only present as phospholipid in cell membranes. Therefore, glycerol quantified intracerebrally is used as a measure of brain cell damage and death [5]. This damage may be caused, for example, by direct mechanical damage and metabolic distress, including inflammation. Because the BBB may be damaged by the injury, it is important to reduce further damage to the BBB and to avoid treatment measures that may worsen the leakage through the BBB [6, 7]. In case of a disrupted BBB, there may be an increased transfer into the brain of blood-borne glycerol released from extracranial sources, such as adipose tissue. There is some evidence that peripheral glycerol may penetrate into the central nervous system after brain injury [8]. If this occurs, the value of glycerol as a marker of cerebral tissue injury could be questioned.

Osmotherapy is one of the most commonly used emergency treatment measures in patients with sTBI with high intracranial pressure (ICP). Intraarterial administration of mannitol may open the BBB [9]. Whether this is true for intravenous administration of mannitol, in doses used in the clinical management, leading to influx of peripheral constituents in the brain, is not clear. If osmotherapy would further open an already damaged BBB, allowing blood constituents to penetrate the brain, measurement of, for example, brain extracellular glycerol, would be hard to use as a marker of brain damage. Surprisingly, there are almost no publications reporting on any correlation between cerebral and other sources of glycerol measured temporally.

Brain tissue damage can be quantified on the basis of levels of protein S-100B and neuron-specific enolase (NSE) in the peripheral blood. S-100B is a calcium-binding protein that is mainly found in the glial cells in the central and peripheral nervous system. Measured in blood, it has an estimated sensitivity of 96% and a specificity of 31% to detect intracranial injuries seen on computed tomography (CT) scans [10]. NSE is a glycolytic enzyme found in the cytoplasm of neurons but also in erythrocytes and platelets. It has shown a varying ability to detect brain injuries seen on CT scans [10, 11].

Increased ICP has been shown to be a prognostic factor for poor outcome after TBI [12]. At the university hospital in Umeå, a modified Lund concept is used in the treatment of TBI. The guidelines used in the Lund concept are focused on volume regulation of the brain and microcirculation and aim at controlling ICP and optimizing microcirculation in the penumbra zone. The goal is to reduce or prevent secondary injury, such as ischemia and edema [13, 14].

We hypothesized that cerebral glycerol (glycerol_brain_) levels would not be associated with peripheral subcutaneous adipose glycerol (glycerol_sc_) levels. If a correlation was observed, this might call into question the use of brain glycerol as a solid marker of brain tissue damage not mirrored in the brain CT classification, biochemical biomarkers, and clinical outcome. The first aim was to study the relationship between intra- and extracerebral glycerol (glycerol_brain/sc_) levels. The second aim was to study any associations between glycerol_brain/sc_ and measurements of brain tissue damage, overall injury level, injury type, and clinical outcome.

## Methods

### Patients

Data were collected from a prospective randomized double-blinded study of prostacyclin versus a placebo [15]. The inclusion criteria were verified severe traumatic head injury, a Glasgow Coma Scale Score (GCS) of 8 or less at intubation and sedation, age between 15 and 70 years, a first measured cerebral perfusion pressure (CPP) of 10 mm Hg or higher, and arrival at our unit within 24 h of trauma. Exclusion criteria were pregnancy or women who were breastfeeding, known bleeding disorder, known allergy to epoprostenol, and penetrating head injury. If the need for intensive care and intubation lasted for less than 72 h in patients discharged from the intensive care unit alive, the injury was considered too mild to be characterized as sTBI, even if the patient initially has a GCS ≤ 8. These patients were excluded. All patients were treated in accordance with the modified Lund concept [13, 15].

**Fig. 1:**
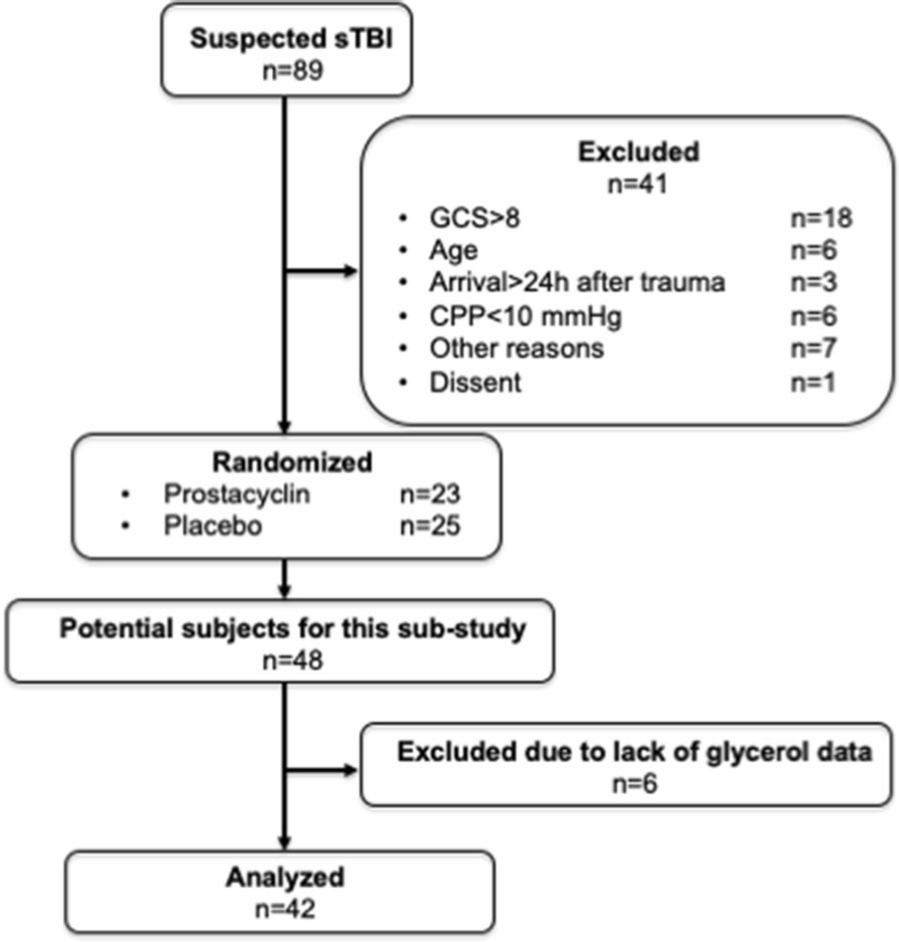
A schematic presentation of the inclusion and exclusion of the patients. CPP cerebral perfusion pressure, GCS Glasgow Coma Scale Score

In the primary study, 48 patients were included [15]. In this retrospective analysis of prospectively collected data, six patients were excluded because of lack of data regarding concomitant glycerol levels in the brain and subcutaneous tissue (see Fig. 1). Thus, 42 patients were included. Demographics and basic information are given in Table 1.

**Table 1:** Demographics and general parameters in relation to brain/subcutaneous glycerol groups

	Age (years), mean ± SD	Sex	GCS, median (range)	ISS, median (range)	APACHE II, median (range)	MAP (mm Hg), mean ± SD	ICP (mm Hg), mean ± SD	CPP (mm Hg), mean ± SD	Rotterdam_init_, median (range)	Catheter tip distance to lesion (mm), mean ± SD
Total (*N* = 42)	37.1 ± 15.2	Women = 15, men = 27	6 (3–8)	29 (9–50)	20.5 (14–32)	81.0 ± 6.0	17.5 ± 9.8	62.5 ± 12.9	3 (1–5)	15.6 ± 12.3
Positive correlation (*n* = 25)	34.2 ± 14.8	Women = 10, men = 15	6 (3–8)	29 (9–50)	19 (14–32)	82.0 ± 6.5	18.7 ± 12.4	61.5 ± 16.4	3 (1–5)	15.5 ± 13.7
Negative correlation (*n* = 17)	41.4 ± 15.2	Women = 5, men = 12	6 (3–7)	29 (16–34)	21 (14–30)	79.6 ± 4.9	15.8 ± 3.5	64.0 ± 4.7	4 (1–4)	15.8 ± 10.8

Basic parameters of the total material and divided into those with a positive or negative correlation between cerebral and subcutaneous adipose glycerol levels

APACHE II, Acute Physiology and Chronic Health Evaluation Score, CPP, cerebral perfusion pressure, GCS, Glasgow Coma Scale Score, ICP, intracranial pressure, ISS, Injury Severity Score, MAP, mean arterial blood pressure, Rotterdam_init_, Initial Rotterdam score

### Monitoring

In all patients, an intraparenchymal ICP-measuring device (Codman MicroSensor, Johnson & Johnson Professional, Raynham, MA) was inserted through a burr hole, approximately at Kocher’s point, preferably on the right side. ICP was measured continuously. Invasive arterial blood pressure was monitored continuously, and the reference level was set at heart level. Mean arterial blood pressure (MAP) and CPP were calculated automatically by the monitoring equipment (Marquette Solar, General Electric Medical Systems, Milwaukee, WI) by using the systolic and diastolic blood pressures registered. The reference point for ICP and MAP was at the same level, resulting in an insignificant hydrostatic difference between the measuring sites. No correction for the CPP calculations was therefore needed.

### S-100B and NSE

Blood samples for biomarkers S100-B and NSE were collected at arrival, and thereafter sampling was repeated at 12-h intervals during the first five days. Centrifugation and freezing of serum in a − 70 °C storing freezer was performed immediately after sampling. Analyses were performed with the automatized LIAISON system by using the LIAISON Sangtec 100 assay for analyses of S-100B and the LIAISON NSE assay for the analysis of NSE (AB DiaSorin, Sangtec Medical, Bromma, Sweden). The highest (maximum) levels of S-100B and NSE, as well as the bulk release during the first 72 h, were used in the calculations because we have previously shown that this provides higher sensitivity in describing the injury [16].

### Microdialysis

Two microdialysis catheters (CMA 70, CMA Microdialysis, Solna, Sweden) with gold tips were placed intracerebrally on each side frontally, approximately at Kocher’s point. Catheter A corresponded to the most injured hemisphere, close to the penumbra zone, assessed through the initial CT scan. Catheter B corresponded to the other hemisphere. Another microdialysis catheter, Catheter C (CMA 60, CMA Microdialysis, Solna, Sweden) was placed in the subcutaneous adipose tissue on the upper part of the abdomen.

The flow rate in all three catheters was 0.3 mL/minute, and the CMA 106 and CMA 107 microdialysis pumps (CMA Microdialysis, Solna, Sweden) were used. For the brain catheters, perfusion fluid CNS (CMA Microdialysis, Solna, Sweden) was used, and perfusion fluid T1 (CMA Microdialysis, Solna, Sweden) was used for the subcutaneous catheter.

The first dialysate was discharged to avoid falsely elevated levels of glycerol due to the minor lesion that could be introduced by the less than 1 mm in diameter microdialysis catheter. Sampling was started 0.5–2.5 h after microdialysis was initiated. Samples were collected for 2 h each over the course of 96 h, resulting in approximately 48 samples per patient. The samples were analyzed by using a CMA 600 analyzer (CMA Microdialysis, Solna, Sweden). If not immediately analyzed, the vials were stored in a freezer (− 18 °C) for a maximum of 24 h and thereafter frozen to − 70 °C. The microdialysis data were transferred from the CMA 600 analyzer to a computer and further processed with LABpilot software (CMA Microdialysis, Solna, Sweden).

In this publication, we report on the absolute glycerol levels from the catheter in the most injured hemisphere (glycerol_brain_) and subcutaneous adipose tissue (glycerol_sc_). For comparison, we also report the glucose (glucose_brain_), lactate (lactate_brain_) levels, and the lactate/pyruvate ratio in the brain measured in the same samples as glycerol. The distance from the microdialysis catheter tip to the lesion (hematoma, evacuated hematoma, or contusion) in the brain was calculated from CT scans. In case of diffuse injury, without evacuated hematomas or contusions, the catheter tip was in the edematous tissue and therefore not included in the calculated distance.

The patients were dichotomized into having a positive or negative correlation between cerebral and subcutaneous adipose glycerol levels. A positive correlation was defined as a linear regression plot with a positive slope; a negative correlation, if the slope was negative.

### CT Scan Evaluation

The first CT scan of the brain and the scan 24 h after the injury were classified on the basis of the Rotterdam scoring system [10, 17]. The Rotterdam scoring system is used to evaluate CT scans of a damaged brain and to estimate prognosis after TBI. Scores are given on the basis of certain criteria, such as compression of basal cisterns, midline shift, traumatic subarachnoid hemorrhage, intraventricular hemorrhage, and epidural hematoma [18].

### Injury Severity and Type of Injury

The Injury Severity Score (ISS) was used for evaluation of the overall injury severity, and the Acute Physiology and Chronic Health Evaluation Score II (APACHE II) was used for measurement of the severity of illness. Type of injury was reported as fall, sports, traffic accident, or assault.

### Outcome

The outcome 6 months post injury was evaluated by an independent staff member using structured interviews and the Glasgow Outcome Score Extended (GOSE) scale. The GOSE is a scale ranging from 1 to 8 points, corresponding to a range of outcomes from dead (1 point) to full recovery (8 points) [19].

### Statistics

Continuous variables are reported as mean ± standard deviation, and median (range) is given for discrete variables. Statistical analysis was performed by using the software JMP 14.2.0 (SAS Institute, Inc., Cary, NC). The χ^2^ test was used for evaluation of differences between proportions. The two-tailed unpaired *t*-test was used for comparisons of differences between groups with continuous variables. Wilcoxon’s rank-sum test was used for discrete variables and for comparison of S-100B and NSE in relation to glycerol because the S-100B and NSE levels varied. Correlation analysis was performed with Pearson’s linear method or Spearman’s *ρ*.

### Ethics and Approval

The Regional Board of Ethics at Umeå University (00–175) and the Swedish Medical Products Agency (151:633/01) approved the study. The study is registered as a clinical trial (ClinicalTrials.gov identifier NCT01363583).

## Results

Demographics and general data are shown in Table 1. All patients had sTBI and a GCS ≤ 8 and were intubated and in need of neurointensive care. The distances from microdialysis catheter tip to lesion are presented in Table 1. Types of accidents were traffic accidents, falls, sports, and assaults (see Table 2). No significant difference between the groups was observed.

**Table 2:** Types of accidents in relation to brain/subcutaneous glycerol groups

Injury type, *n* (%)	Total (*N* = 42)	Positive correlation (*n* = 25)	Negative correlation (*n* = 17)
Traffic accident	28 (66.7)	19 (76)	9 (52.9)
Fall	10 (23.8)	4 (16)	6 (35.3)
Sport	1 (2.4)	1 (5)	0
Assaults	3 (7.1)	1 (5)	2 (11.8)

Types of accidents in the total material and divided into those with a positive or negative correlation between cerebral and subcutaneous adipose glycerol levels

**Fig. 2:**
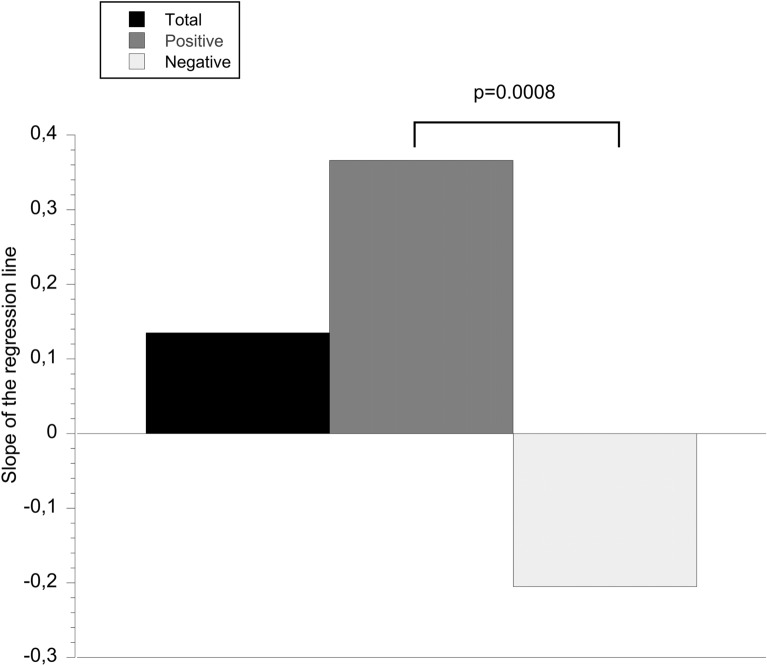
Bar plot of the regression plot slope coefficient between brain and subcutaneous glycerol in the total group and groups with a positive or negative correlation. Two-tailed unpaired *t*-test

**Fig. 3:**
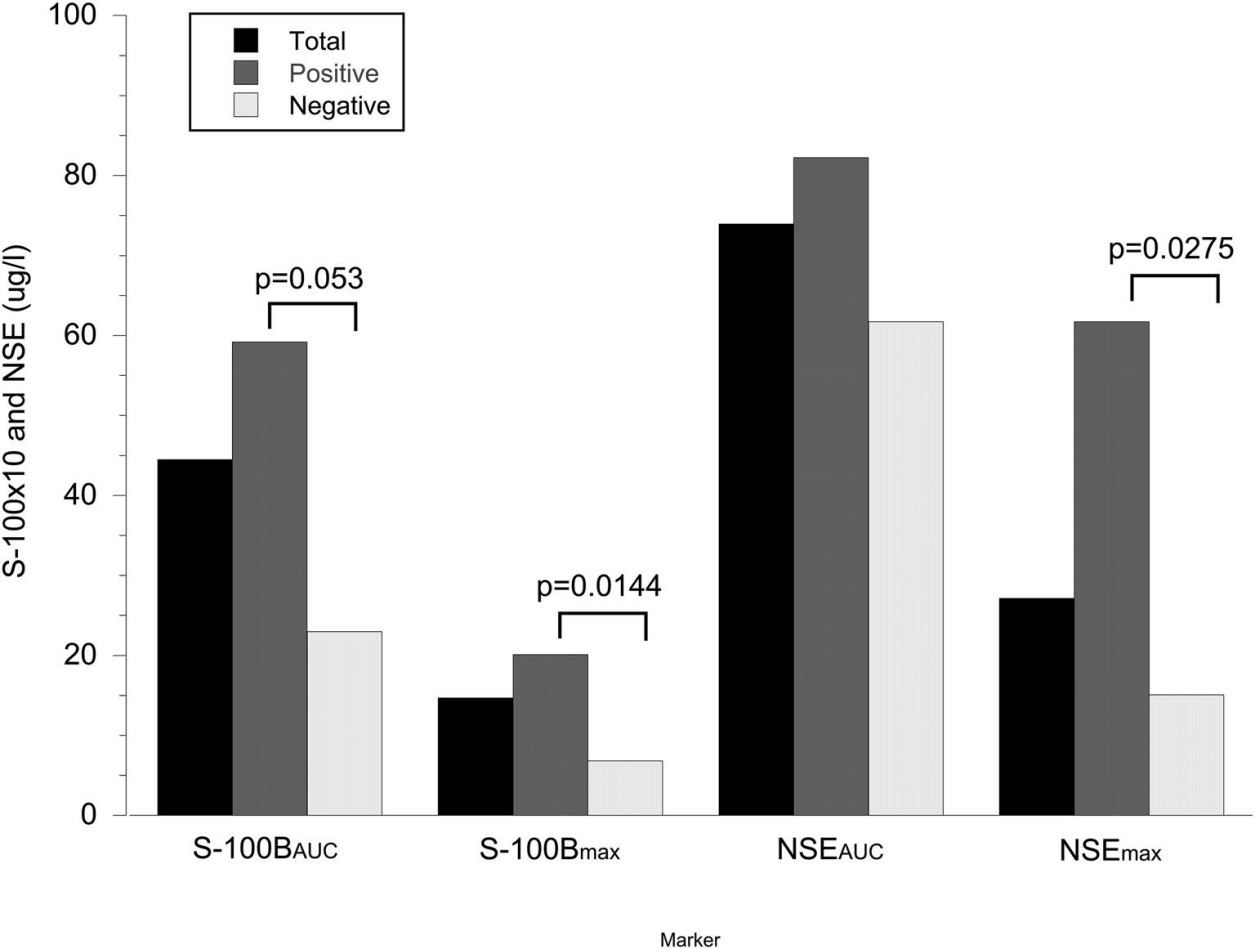
Bar plot of the levels of the biochemical biomarkers in relation to the regression plot slope coefficient between brain and subcutaneous glycerol in the groups with a positive or negative correlation. Observe that the S-100B values are × 10 for visibility. Two-tailed unpaired *t*-test. NSE_AUC_ bulk release of neuron-specific enolase, NSE_max_ maximal level of neuron-specific enolase, S-100B_AUC_ bulk release of S-100B, S-100B_max_ maximal level of S-100B

There were 25 individuals with a positive glycerol_brain/sc_ correlation (individual Pearson’s *R*^2^ coefficients between 0.0002 and 0.4144) and 17 with a negative correlation (individual Pearson’s *R*^2^ coefficients between 0.000001 and 0.4610). The corresponding regression plot results were significantly different (Table 3). Figure 2 summarize these results. Prostacyclin had no significant effect on the association of glycerol_brain/sc_ in either group (*t*-test). The mean value of glycerol_brain_ in the first 96 h was slightly higher in the group with a positive correlation of glycerol_brain/sc_ compared with the group with a negative correlation (Table 3). These results are summarized in Fig. 3. There was no significant difference in glycerol_sc_ levels between groups with positive or negative correlation of glycerol_brain/sc_.

**Table 3:** Biomarkers in relation to brain/subcutaneous glycerol groups

	Slope of the regression line, mean ± SD	S-100B_AUC_ (µg/L), mean ± SD	S-100B_max_ (µg/L), mean ± SD	NSE_AUC_ (µg/L), mean ± SD	NSE_max_ (µg/L), mean ± SD	Glycerol_brain_ mean 96 h (µg/L), mean ± SD	Glycerol_sc_ mean 96 h (µg/L), mean ± SD	Glucose_brain_ mean 96 h (mmol/L), mean ± SD	Lactate_brain_ mean 96 h (mmol/L), mean ± SD	Lactate/pyruvate, mean ± SD
Total (*N* = 42)	0.135 ± 0.633	4.45 ± 10.2	1.47 ± 3.33	73.94 ± 85.98	27.15 ± 35.01	127.05 ± 143.65	234.38 ± 106.08	1.77 ± 1.10	3.73 ± 1.94	45.55 ± 30.00
Positive correlation (*n* = 25)	0.366 ± 0.719	5.92 ± 13.1	2.01 ± 4.26	82.23 ± 90.91	35.37 ± 4.26	137.03 ± 112.20	225.82 ± 91.67	1.74 ± 1.18	3.57 ± 1.63	47.81 ± 35.03
Negative correlation (*n* = 17)	− 0.205 ± 0.216^a^; *p* = 0.0008	2.30 ± 1.34^b^; *p* = 0.053	0.68 ± 0.36^b^; *p* = 0.0144	61.74 ± 76.25	15.08 ± 10.61^b^; *p* = 0.0275	111.45 ± 185.62	246.97 + 126.28	1.81 ± 1.00	3.96 ± 2.34	42.03 ± 20.36

Slope of the regression line and biochemical biomarker levels in the total material and in the patients with a positive or negative correlation between cerebral and subcutaneous adipose glycerol levels

Glucose_brain_, brain glucose level, Glycerol_brain_, brain glycerol level, Glycerol_sc_, subcutaneous glycerol level, Lactate_brain_, brain lactate level, NSE_AUC_, bulk release of neuron-specific enolase, NSE_max_, maximal level of neuron-specific enolase, S-100B_AUC_, bulk release of S-100B, S-100B_max_, maximal level of S-100B

^**a**^*t*-test, comparison between positive and negative correlation

^b^Wilcoxon’s rank-sum test, comparison between positive and negative correlation

There was no significant difference in mean age (*t*-test) or GCS (Wilcoxon’s rank-sum test) in the two groups (see Table 1).

No significant association between initial CT Rotterdam score or that after 24 h and glycerol_brain/sc_ (Wilcoxon’s rank-sum test) (Table 1) was observed. There was a positive correlation between the Rotterdam score at 24 h after injury (but not the initial score) and mean glycerol levels in the brain (*ρ* = 0.3565, *p* = 0.0221, Spearman). No such correlation was observed with the peripheral glycerol levels. There was a significant association of S-100B_max_, S-100B_AUC_, NSE_max_, and glycerol_brain/sc_ (Wilcoxon’s rank-sum test) (Table 3). We found no association between other brain metabolic markers (glucose, lactate, lactate/pyruvate) and the glycerol_brain/sc_ patterns (Table 3).

MAP (mean 96 h) did not differ between the two groups. No association between ICP or CPP levels (mean 96 h) and glycerol correlation was observed (Table 1).

There was no difference in the proportion of decompressive hemicraniectomies between the groups (χ^2^ test).

Type of injury, ISS, and APACHE II had no impact on the results, and there were no significant differences in these parameters between the groups (Table 1), nor was there any correlation between the brain and peripheral glycerol levels or between the ratio of brain to peripheral glycerol and ISS and APACHE II.

At the 6-month follow-up, the median GOSE was 4 (1–8) in the group with a negative glycerol_brain/sc_ correlation and 5 (1–7) in the group with a positive correlation (ns), Wilcoxon’s rank-sum test).

## Discussion

We have shown that in 60% of patients with sTBI, peripheral glycerol may flux into the brain. This effect is associated with worse brain tissue damage, as measured by S-100B and NSE.

Glycerol has been considered as a marker for brain cell damage after TBI [5] and may be correlated with clinical outcome. This damage may be a direct mechanical damage or damage due to metabolic distress. In a situation with a flow of glycerol from the blood into the brain due to, for example, a BBB disruption, the intracerebral glycerol level ceases to mirror the intracerebral condition. In such a situation the glycerol levels in the brain may be misinterpreted to indicate an increased breakdown of brain tissue. In 60% of the patients, a positive correlation was observed. This may be interpreted as there being a possibility of an inflow of glycerol to the brain and may be due to, for example, some kind of BBB dysfunction. Therefore, one might suggest that some caution should be used in interpreting brain interstitial levels of glycerol as a marker of brain cell damage. The pathophysiological mechanism behind the negative correlation between extra- and intracerebral glycerol levels is interesting. One reason for this pattern could be that the peripheral glycerol level mirrors extracranial injuries, resulting in an altered glycerol ratio between peripheral and intracranial tissue. However, we could not identify any difference in type of accident, extracranial injuries, or level of injury severity, as measured by using ISS and APACHE II, nor was there a difference in peripheral glycerol levels between the groups. In addition, we found no indication of a difference in the metabolic patterns in the brain, as reflected in the interstitial concentrations of glucose, lactate, and lactate/pyruvate.

All patients in this study were treated with β-blockers (metoprolol) and α_2_-stimulation (clonidine) to decrease the sympathetic nervous system drive, thus decreasing the general stress level and by extension, the inflammation process. Because subcutaneous glycerol release is promoted by an activation of the sympathetic nervous system, and we were actively trying to decrease this response, it is unlikely that the general stress levels of the patients influenced the results. We did not measure the blood level of glycerol, but it has previously been reported that subcutaneous glycerol levels measured by microdialysis follows the pattern of glycerol in the blood [20].

There was no significant association of ICP or CPP and glycerol_brain/sc_. This can simply reflect effects of the treatment striving to normalize the ICP and CPP. It is more remarkable that the tissue damage categorized by using the Rotterdam score was not associated with extra- to intracranial glycerol correlations. However, surrogate measures of brain tissue damage, i.e., S-100B and NSE, showed more impairment in the patients with a positive correlation of glycerol_brain/sc_. This may indicate that the greater the brain tissue damage, the greater the leakage of glycerol from extracerebral tissues (subcutaneous fat) into the brain, which may be due to a disrupted BBB.

We have previously shown that prostacyclin treatment is associated with a stabilization of the pressure reactivity of cerebral vessels [21] and the relation between cerebral glycerol and pressure reactivity in sTBI [22]. Interestingly, in the present study, prostacyclin had no clear-cut influence on the intra- to extracerebral glycerol correlation. Thus, one could speculate, that the previously reported effect of prostacyclin on pressure reactivity and glycerol is not influenced by an extra- to intracerebral glycerol flux.

Some support for our findings may be found in the publication by Meyer et al. [8]. They showed, in patients with cerebral infarction, that intravenous infusion of glycerol entered the brain and cerebrospinal fluid. In addition, glycerol given for treatment of high ICP in patients with brain edema after a large middle cerebral artery infarction resulted in a marked accumulation of brain interstitial glycerol, as measured through microdialysis [23]. To the best of our knowledge, no previous studies have been reported on de novo intra- to extracranial glycerol correlation in sTBI. Thus, it is impossible to compare our results with others. Because glycerol is clinically used as a very potent hyperosmotic drug in patients with life-threatening intracerebral processes, it is wise to consider that a disrupted BBB may cause leakage of glycerol into the brain tissue. One could speculate that hyperosmotic agents clinically used in attempts to reduce brain edema in patients with sTBI may further increase such leakage, leading to an increased osmotic tissue pressure. This may result in an aggravated tissue edema, a phenomenon that can be observed in these severely ill patients.

As with all clinical and experimental studies, there are strengths and weaknesses. The strengths of our study are that all patients had been treated and monitored in accordance with a predefined schedule. All patients were from a specified region and havd sTBI. Weaknesses include that we only studied patients with sTBI and therefore our results cannot directly be applied on patients with mild and moderate head injury. In addition, whether our results are applicable on patients treated by different treatment algorithms is unknown. Our study is too small to draw any certain conclusions. Therefore, further studies must validate or reject the present results.

## Conclusions

There may be an influx of glycerol from extracerebral sources into the brain, confusing the interpretation of the cerebral glycerol levels. This eventual influx was paralleled with higher S-100B and NSE levels, indicating a worse brain tissue injury. We remind the clinicians and researchers that a damaged BBB, as seen in sTBI, may alter the levels of various substances, including glycerol in the brain. Awareness of this is important in the interpretation of the bedside data as well as in research.
